# Salivary cortisol response to post-exercise infrared sauna declines over time

**DOI:** 10.1080/23328940.2025.2493460

**Published:** 2025-04-27

**Authors:** Essi K. Ahokas, Heikki Kyröläinen, Johanna K. Ihalainen, Helen G. Hanstock

**Affiliations:** a Faculty of Sport and Health Sciences, University of Jyväskylä, Jyväskylä, Finland; b Finnish Institute of High Performance Sport KIHU, Jyväskylä, Finland; c Department of Health Sciences, Swedish Winter Sports Research Centre, Mid Sweden University, Östersund, Sweden

**Keywords:** Heat-therapy, recovery, stress response, autonomic nervous system, female athletes

## Abstract

Heat exposure after exercise may enhance recovery of physical performance but can also impose additional physiological stress on athletes. This study investigated the effects of post-exercise infrared sauna (IRS) on adrenal and autonomic nervous system (ANS) responses and examined how these responses adapt over time during a 6-week training intervention. Forty female team-sport athletes were pair-matched into an IRS-group and a control group (CON). Participants completed jumping exercises followed by IRS (10 min, 50 °C) or passive recovery and physiological assessments during two experimental trials: in the first (EX1) and in the last (EX2) week of the training intervention. The ANS responses were assessed by nocturnal heart rate (HR) and heart rate variability recorded before and after exercise session. Saliva cortisol concentrations, muscle soreness, and perceived recovery were assessed in the morning, before and after the exercise sessions. Cortisol increased by 5.1 ± 8.6 nmol/l the morning after EX1 in the IRS-group (*p* = 0.017), but not in the CON-group. Furthermore, a greater pre-post change in cortisol concentration was observed following EX1 (4.6 ± 10.4 nmol/l) compared to EX2 (−1.8 ± 7.6 nmol/l). The IRS-group showed a higher post-session HR in EX1 compared to the CON-group (61 ± 8 bpm vs. 55 ± 6 bpm; *p* = 0.019). Increased muscle soreness was observed at EX1 post36h only in the CON-group. Post-exercise IRS initially elevated physiological stress responses in female athletes. After six weeks of regular IRS use, athletes’ ANS balance and cortisol response adapted, suggesting effective physiological adjustment to the heat intervention within six weeks.

## GRAPHICAL ABSTRACT

**Figure:**
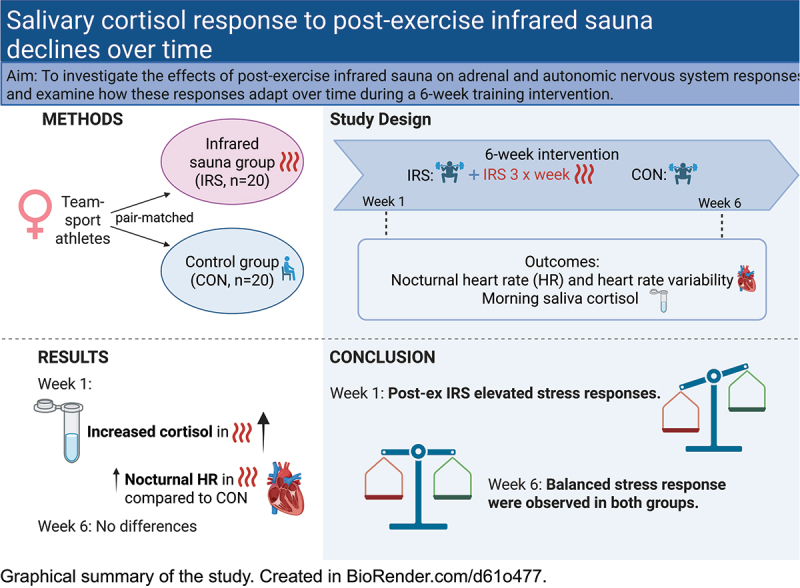

## Introduction

Athletes commonly use exposure to heat through saunas, hot baths or other methods after exercise as a strategy to promote recovery of physical performance. However, contradictory findings remain in terms of whether post-exercise heat exposure is beneficial for recovery. While some studies have shown positive effects [1–3], others suggest that post-exercise heat exposure could serve as an additional source of physiological strain and thus be detrimental to recovery [4,5].

From the perspective of the allostatic load model, it is reasonable to hypothesize that the addition of heat to the recovery regime may inadvertently increase physiological strain and delay recovery. Allostatic load is the cost to the body of constantly adapting to repeated or chronic stressors [6]. Increases in allostatic load can be associated with dysregulated stress responses, with stress-mediating mechanisms – such as the sympathetic nervous system and the hypothalamic – pituitary – adrenal (HPA) axis responding disproportionately to stressors [6]. In this context, athletes’ internal load, fatigue, and potential overreaching are often assessed via stress hormone levels, such as morning cortisol levels or acute cortisol responses to stress or exercise – known as cortisol reactivity [7], or by using autonomic nervous system (ANS) markers, such as nocturnal heart rate variability (HRV) [8].

The neuroendocrine system is considered one of the most responsive physiological systems to stressors and serves as a key signaler, helping other systems adapt to stress and restore homeostasis [9]. Passive heat exposure without a prior exercise session has been found to induce variable blood cortisol responses [10]. However, higher air temperatures and longer exposure durations appear to evoke greater cortisol responses [10]. In addition, it is well established that unaccustomed heat exposure augments stress hormone responses to exercise [9], but that the response is attenuated after heat acclimation [11,12], indicating that athletes generally have a good capacity to adapt positively after incorporating heat exposure as an additional stressor during their training.

In tandem with neuroendocrine responses, autonomic regulatory pathways play a crucial role as mediators of physiological stress. Heat exposures, particularly traditional sauna bathing, have been shown to be beneficial for cardiovascular health, including ANS function, both alone and in combination with exercise [13]. Notably, passive heating has been found to shift autonomic cardiovascular control from vagal dominance toward greater sympathetic control [14,15]. Furthermore, post-exercise infrared sauna (IRS) and traditional sauna have been shown to decrease vagal tone during heat exposure [1,16]. However, one study has shown that post-exercise IRS did not affect nocturnal HRV during the recovery period [1]. Additionally, studies on repeated heat exposure and resulting heat acclimation have yielded mixed findings regarding their effects on HRV responses [16–18].

Although existing research on post-exercise heat exposure suggests that it might increase stress reactivity to exercise, it is important to note that most studies to date have predominantly involved male participants [1,12,14,16–20]. However, neuroendocrine and ANS responses to passive heating can differ between men and women, with women exhibiting a pronounced hormonal response to heat [15] and a reduced very-low frequency HRV response to cold environment [21]. Thus, stress responses to training followed by heat exposure should also be studied in female participants. Nevertheless, the menstrual cycle phase and the use of hormonal contraceptives can cause fluctuations in body temperature, which may affect thermoregulation [22]. Additionally, exercise-induced stress responses are known to decrease with regular training [9] and physiological responses to exercise may differ between recreationally active participants and competitive athletes [23], which is why it is important to study these responses in athletes.

Therefore, this study aimed to investigate the effects of post-exercise IRS exposure on stress responses of female team-sport athletes in a practical, real-world setting. Jump training, including ballistic and plyometric exercises, has been shown to enhance neuromuscular performance and power output in team sport athletes [24,25]. Unlike traditional strength training, the concentric phase of ballistic exercises does not involve a deceleration phase, but instead acceleration continues throughout the movement. Movement patterns include a stretch-shortening cycle [26], and power output remains higher during training [27]. While ballistic training includes weightlifting movements, single jumps, and throws, plyometric training consists of jumping and hopping exercises that involve rapid stretch-shortening cycles and short ground contact times [28,29]. Given the importance of these training modalities for athletic performance, understanding how post-exercise IRS influences stress responses and recovery in this context is crucial. Specifically, we set out to investigate: (1) the acute impact of IRS on stress hormones and ANS responses, (2) effects on subjective muscle soreness and perceived recovery, and (3) how these responses adapt over time with continued post-exercise IRS use during a 6-week training intervention.

## Methods

### Participants

Forty female team sport athletes participated in the study; 33 athletes completed the full study protocol according to the instructions (supplementary Figure 1). Participants were recruited from local basketball (two teams, *n* = 14), futsal (two teams, *n* = 18), ice hockey (*n* = 4) and American football clubs (*n* = 4). Performance level ranged from the highest to 3rd highest leagues (Tier 2–3 in the classification on a scale of 0–5, according to McKay et al. [30]). Prior to inclusion in the study, participants were informed about the study purpose and testing procedures. Written informed consent was obtained from all participants. For those under 18 years of age, written consent was also obtained from a guardian. Additionally, participants completed a health screening questionnaire. The study was conducted in accordance with the Declaration of Helsinki (2013), except for pre-registration in a database, and ethical approval was provided by the Ethics Committee of the University of Jyväskylä (1516/13.00.04.00/2021).

**Figure 1.**
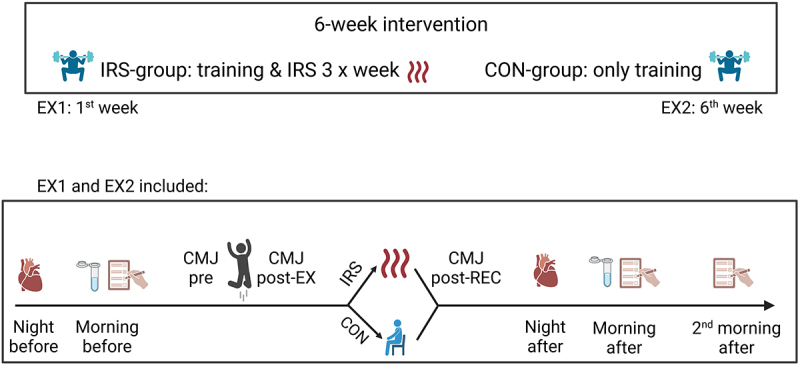
Schematic view of the study design. Participants completed a standardized exercise session protocol (jumping person) followed by sitting in an infrared sauna (IRS, heat waves) or in room temperature (person in the chair) during the first and the sixth week of the intervention. Heart rate and heart rate variability (heart), saliva samples (saliva tube), and subjective measures (muscle soreness and perceived recovery; questionnaire) were assessed before and after the session. *CMJ* countermovement jump; *EX1* exercise session 1; *EX2* exercise session 2; *pre* before the exercise protocol, *postEX* after the exercise protocol; *postREC* after the recovery method. Created in BioRender.com/b34o251.

Athletes were pair-matched into two groups, an IRS group (IRS, *n* = 20, age: 22 ± 5 yrs.; height: 167 ± 7 cm; body mass: 69.4 ± 18.2 kg) and a control group (CON, *n* = 20, age: 23 ± 5 yrs.; height: 168 ± 6 cm; body mass: 69.5 ± 13.7 kg), with the matching process prioritizing team affiliation, followed by neuromuscular performance (20 m sprint, counter movement jump (CMJ), maximal voluntary contraction), and age. Groups were evenly allocated within teams. Sauna usage history was assessed through a questionnaire on sauna bathing habits. Three participants (IRS: 1, CON: 2) reported no sauna use in the past six months. Twenty-one participants (IRS: 11, CON: 10) indicated sauna bathing approximately once a month, while eight (IRS: 5, CON: 3) reported weekly use. Six participants (IRS: 3, CON: 3) sauna bathed 2–5 times per week. One participant had experienced an IRS session once, whereas the others had never used it. Two participants did not return the completed questionnaire.

All participants were instructed to not consume any alcohol, and to refrain from any additional exercise and heat exposures 24 hours prior to the experimental trials and on the day of experimental trials. Additionally, it is known that sex hormone fluctuations can influence cortisol reactivity [9]. Thus, even though it can be assumed that minimal hormonal profile changes occurred during the 36 hours of each exercise trial, a menstrual diary was completed throughout the training intervention. The use of hormonal contraceptives, manifestation of amenorrhea, oligomenorrhea (among naturally menstruating participants), as well as cycle phase during the exercise sessions are presented in supplementary table 1.

## Study design

The study was part of a parent project investigating the regular use of post-exercise IRS on training adaptations [31]. To investigate the acute stress responses to strength and power training followed by heat exposure (IRS group) or training alone (control group), participants completed a standardized neuromuscular exercise session with various jumping exercises, along with physiological measurements. Immediately after the session, participants in the IRS-group sat in the IRS for 10 min while participants in CON sat in the room temperature. To observe how stress responses adapt over time with continued use of post-exercise IRS, two experimental trials were scheduled during the first and last weeks of a 6-week strength and power training intervention (termed exercise session 1 and 2, EX1 and EX2, respectively; Figure 1).

During the intervention, participants performed strength and power training 2–3 times per week. Exercises were selected based on sport-specific demands and paired to combine resistance and explosive movements, performed at maximal speed. Training volume and intensity progressed over six weeks, starting at 70–80% of one-repetition maximum (3 sets), increasing to 80–85% (4 sets), and then tapering to 50–55% (3 sets), with repetitions in reserve used for load adjustments. The exercises and the repetitions are shown in supplementary table 2. Athletes continued their teams’ regular sport-specific and endurance training but were instructed to avoid engaging in additional recovery strategies outside their assigned intervention.

Neuroendocrine and ANS responses to stress, influenced by training followed by heat exposure or training alone, were measured over two days during each trial. The ANS responses were assessed via nocturnal heart rate (HR) and HRV measured during the nights before (pre) and after (post) the exercise session. Neuroendocrine responses via saliva stress hormone concentrations were assessed in the morning, both before and after the exercise session. To analyze the effects of IRS on subjective muscle soreness and perceived recovery, participants completed questionnaires in the morning before and after the exercise session. Furthermore, neuromuscular performance was assessed via CMJ-test immediately before (pre) and immediately after (post-EX) the exercise session, and immediately after the recovery (post-REC) to determine that exercise session induced similar fatigue in both groups and to investigate immediate effects of post-exercise IRS on neuromuscular performance. Participants were familiarized with the use of HRV-monitors, questionnaires, and saliva sampling over 2–3 nights and mornings, around a week before the first trial.

## Neuromuscular exercise session

Participants performed a standardized training session consisting of jumping exercises twice, with a five-week interval between the sessions. The exercises included both ballistic and plyometric drills and were modified based on previous studies with female team sport athletes [25,32]. After a standardized warm-up, participants performed five jump exercises: 1) 3 sets x 5 repetitions CMJ with additional load (25% body weight), 2) 3 × 4 drop jump (30 cm box), 3) 3 × 5 box jump (50 cm box), 4) 3 × 10 bounding, 5) 3 × 5 double leg hop. Inter-set rest intervals were 2 minutes and rest periods between exercises were 3 minutes.

## Infrared sauna session and passive recovery

The IRS (VitaMy, Sentiotec GmbH, Vöcklabruck, Austria) had two seats with IR-emitting panels at the front and back. The temperature of the sauna was set to 50°C 15–25 minutes before sauna bathing, with 45 (12) % relative humidity (Suomen lämpömittari 7440, Helsinki, Finland).

Participants in the IRS group sat in the IRS for 10 min, wearing sports bras and shorts, starting about five minutes after session completion. The body temperature of the participants during sauna bathing was not measured but was piloted with eight participants (age 30.4 (3.5) years, height 168.3 (6.5) cm, body mass 62.6 (6.1) kg) as described in Ahokas et al. [31]. Tympanic temperature increased from 36.5 (0.3) °C to 37.1 (0.2) °C. Weighted mean skin temperature increased from 33.3 (1.1) °C to 36.4 (0.2) °C. Participants in the control group sat in the room temperature for 10 min.

## Lower body power production

To assess acute lower body maximal power output, a CMJ-test was performed before the exercise session (pre), 3 minutes after completion of the session (post-EX), and 2 minutes after completion of the recovery protocol (post-REC). The CMJ flight time was determined using a contact mat (University of Jyväskylä, Finland). Participants kept their hands on their hips and jumped as high as possible, with three to five attempts to achieve their best performance (1 min rest between attempts). A self-determined range of motion was permitted.

## Saliva collection and cortisol analysis

Participants collected saliva samples at home following an overnight fast of 10 h. The participants rinsed their mouths with water 5 minutes before saliva collection using a validated [33] synthetic swab (Salivette Cortisol, Sarstedt, Nümbrecht, Germany), which has been shown to provide high cortisol recovery and minimal binding [34]. Saliva collection using the swab-based method for cortisol analysis has been found to be reliable, correlating better with serum total and free cortisol than saliva collection using the passive drool method [35]. During saliva sampling, participants were asked to rest for 60–90 seconds, while keeping the cotton-swab collector in their oral cavity. Following collection, samples were stored maximum of five days [36] in the fridge and after that centrifuged at 2 400 rpm for 3 min. Thereafter, saliva samples were stored frozen (−20 °C) until analysis. Saliva cortisol concentration was analyzed by validated [37] chemiluminescence immunoassays (IMMULITE 2000 XPi, Siemens Healthcare Diagnostics, UK) using hormone-specific immunoassay kits (Siemens, New York City, USA). The inter-assay coefficient of variation was 8.2% for cortisol.

## Nocturnal heart rate and heart rate variability

A wearable Bodyguard2 sensor (Firstbeat Technologies Ltd, Jyväskylä, Finland) was used to record nocturnal HR and HRV. The accuracy and reliability of the data recorded by the Bodyguard2 sensor for HRV analysis has been shown to be high [38]. Two spot electrodes of the sensor were placed on the skin below the right collarbone and on the left rib cage. The monitor records an electrocardiogram and converts the signal into R-R intervals using an algorithm with an accuracy of 1 ms. Data was transferred from the sensor to the validated [39] Kubios software (Kubios Oy, Kuopio, Finland) for further analysis. Participants wore the monitor while in bed at the night before (pre) and after (post) the exercise session.

Average HR and root mean square of successive differences between normal heartbeats (RMSSD) were analyzed. In addition, power spectral analysis was conducted using a fast Fourier transformation, and the relative values of very low frequency (VLF, 0.01–0.04 hz), low frequency (LF, 0.04–0.15 hz), and high frequency (HF, 0.15–0.40 hz) powers were analyzed. While RMSSD and HF power appear to be the most valid noninvasive measures for assessing parasympathetic nervous system activity, there is conflicting evidence regarding the information that LF and VLF power provides about ANS status [40]. While LF power has been thought to reflect sympathetic nervous system activity, it appears to primarily represent baroreflex-cardiovagal function [40,41]. VLF power, on the other hand, appears to be influenced by the renin-angiotensin system [40], but increases in resting VLF power may also reflect increased sympathetic activity [41].

Participants reported their bedtimes via sleep diaries, which were needed for HR and HRV analyses. The data were analyzed from each night over a four-hour period, commencing 30 min after bedtime. It has previously been reported that when HRV data is averaged over a sufficient period (e.g. 4 hours), nocturnal HRV demonstrated very good day-to-day reliability [42]. Additionally, nocturnal HRV measurements have been shown to be sensitive to training-induced stress and HRV responses associated with performance-related training responses, and nocturnal HRV correlated well with the morning rest HRV measures [43]. The raw HRV data required little artifact correction; the average percentage of corrected beats was 0.4% for both the EX1 and EX2 analyses.

## Muscle soreness and perceived recovery

Participants reported subjective muscle soreness (on a scale of 1–10; 1 = no pain, 10 = very sore [44]) in the morning before (pre) and the two mornings after (post12h and post36h) the exercise session. Additionally, perceived recovery was reported the morning before (pre) and after (post12h) exercise session (on a scale of 1–10; 1 = not recovered at all, 10 = fully recovered).

## Data and statistical analyses

Data were analyzed using IBM SPSS Statistics 28 (IBM Corp. Armonk, NY, USA) and R Studio 2024.09.0 (Posit PBC, Vienna, Austria). Timepoint (pre, post) and group effects, and group*timepoint interactions were analyzed with a linear mixed-effects model. Independent models were created for trials EX1 and EX2. The normality of residuals was checked using the Shapiro-Wilk test, as well as skewness and kurtosis values. Additionally, residuals versus predicted values were visually inspected via scatter plots. If the residuals did not meet normality assumptions or showed issues in the plots, binary logarithm transformed values (saliva cortisol concentrations) were used. When a main effect or interaction was detected, post hoc analyses were performed with Bonferroni adjusted analyses.

Additionally, linear mixed models were used to compare pre-post changes within trials EX1 and EX2, across groups, and to assess trial*group interaction effects. The change between the pre and post was calculated for log_2_-corrected variables as: log_2_ fold change = log_2_(post/pre).

## Results

### Lower body power production

**Figure 2.**
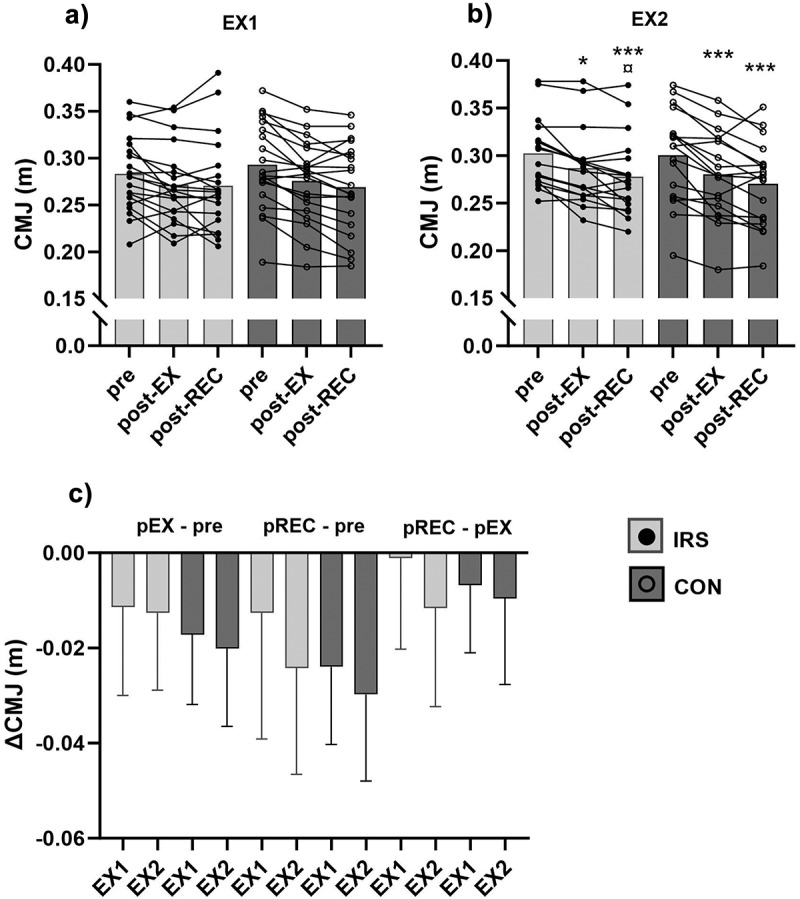
Countermovement jump (CMJ) height immediately before (pre), immediately after the exercise session (post-EX), and immediately after the recovery procedure (post-REC) in (a) the first (EX1) and (b) in the last week (EX2) of training intervention, and (c) the change between timepoints of the CMJ height in EX1 and EX2. *CON* control group, *IRS* infrared sauna group. ***p < 0.001 compared to pre-value, * *p* < 0.05 compared to pre-value, ¤ *p* < 0.05 compared to post-EX.

There were no main (group/timepoint) or interaction effects for CMJ at EX1 (Figure 2(a)). However, a change in CMJ performance over time was observed at EX2, whereby jump height decreased by 1.6 ± 1.7 cm from pre-EX to post-EX (IRS: *p* = 0.017; CON: *p* < 0.001) and 2.7 ± 2.0 cm to post-REC (IRS: *p* < 0.001; CON: *p* < 0.001; Figure 2(b)). In addition, CMJ height (*p* = 0.034) was higher post-EX compared to post-REC in the IRS-group. Although the time effect was found only at EX2, there were no interaction or main effects for the change between timepoints in CMJ height when responses at EX1 and EX2 were compared (Figure 2(c)). Detailed statistics are presented in supplementary tables 3 and 4.

## Cortisol responses

**Figure 3.**
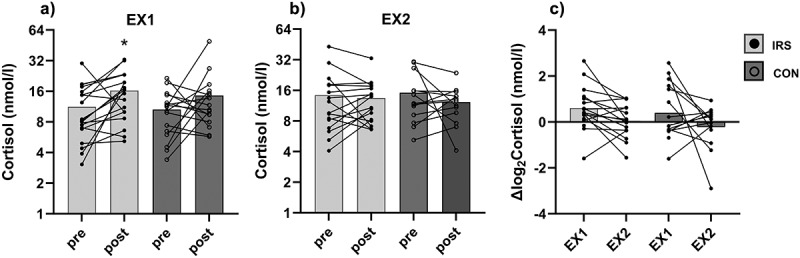
Morning saliva cortisol concentration before (pre) and after (post) the exercise session (a) in the first (EX1) and (b) in the last week (EX2) of the training intervention, and (c) pre-post log_2_ Fold change in cortisol response to session in EX1 and EX2. *CON* control group, *IRS* infrared sauna group. *p < 0.05 compared to pre-value analyzed with log_2_ transformed values.

There were no group x timepoint interactions or group effects for morning saliva cortisol concentration in EX1, but a time effect was observed. After post hoc analyses, a higher morning cortisol concentration was found after EX1 compared to before EX1 in the IRS-group (*p* = 0.017; fold change = 1.52), but not in CON-group (*p* = 0.140; fold change = 1.32; Figure 3(a)). There were no interaction or main effects for saliva cortisol concentration in EX2 (Figure 3(b)).

There was a difference in cortisol responses between the trials. However, post hoc analyses revealed no differences between EX1 and EX2 in both groups (IRS: *p* = 0.100; CON: *p* = 0.095; Figure 3(c)).

## Nocturnal heart rate and heart rate variability

**Figure 4.**
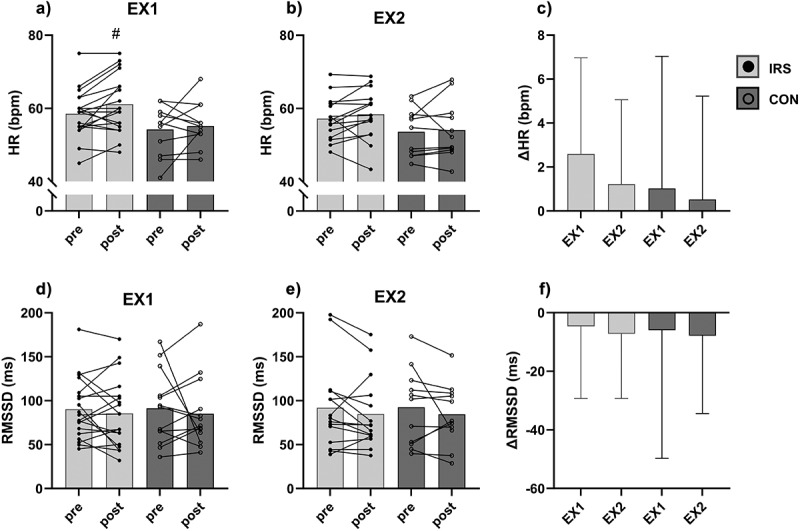
Nocturnal autonomic nervous system variables before (pre) and after (post) the exercise session in the first (EX1) and in the last week (EX2) of the training intervention, and changes in response magnitude in EX1 and EX2 within groups. (a-c) heart rate (HR), (d-f) root mean square of successive differences between normal heartbeats (RMSSD). *CON* control group, *IRS* infrared sauna group. # *p* < 0.05 compared to CON-group.

A group effect was observed for HR in EX1 and EX2, and for VLF in EX2. There were no interaction or time effects for HR and VLF. In post hoc analysis, the IRS-group had higher (*p* = 0.019; 61 ± 8 bpm) HR compared to the CON-group (55 ± 6 bpm) post-EX1, but not pre-EX1 (*p* = 0.086; Figure 4(a)). In EX2, there were no differences between groups in pre (*p* = 0.177) and post (*p* = 0.120; Figure 4(b)). Furthermore, the IRS-group had lower (*p* = 0.028) VLF compared to the CON-group pre-EX1, but not in post-EX1 (*p* = 0.057; Supplementary Figure 2(a)).

There were no interaction or main effects for RMSSD, LF, and HF in EX1 or EX2 (Figure 4(d,e), Supplementary Figure 2(d,e,g-i)). Additionally, there were no interaction or main effects for HR and HRV variable responses to exercise session and recovery (Figure 4(c), Supplementary Figure 2(c,e,j)), when the trials and the groups were compared, indicating similar ANS responses in both trials and groups.

## Muscle soreness and perceived recovery

**Figure 5.**
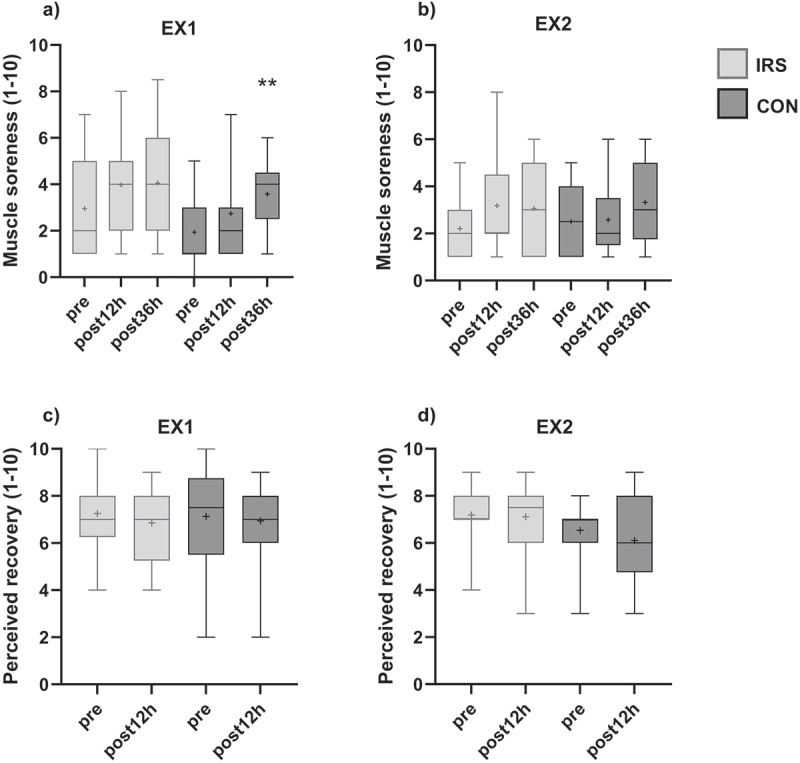
Muscle soreness (a,b) and perceived recovery (c,d) before (pre) and 12 h (post12h) and 36 h (post36h) after the exercise session in the first (EX1) and in the last week (EX2) of training intervention. Mean expressed with +, and whiskers define 5–95%ile. *CON* control group, *IRS* infrared sauna group. **p < 0.01 compared to pre-value.

Group and time effects were found for muscle soreness in EX1 but there was no interaction effect. However, there were no significant differences between the groups in pre (*p* = 0.167), post12h (*p* = 0.072), and post36h (*p* = 0.484) in post hoc analyses. In addition, there were no differences between timepoints in IRS (*p* = 0.102–1.000), but greater muscle soreness was observed at post36h compared to pre in CON-group (*p* = 0.039; Figure 5(a)). There were no main or interaction effects for muscle soreness in EX2.

There were no main or interaction effects for perceived recovery in EX1, but a group effect was observed for perceived recovery in EX2. However, there were no differences between groups in pre (*p* = 0.334) and post12h (*p* = 0.092; Figure 5(c)) in post hoc analyses. In addition, there were no interaction or main effects for the change between timepoints in subjective variables, when the trials and the groups were compared (Figures 5(b,d)).

## Discussion

This study investigated the acute effects of post-exercise IRS on female team-sport athletes’ stress responses during the first and the last week of a 6-week intervention combining strength and power training and post-exercise IRS. In the first week of the training intervention, some indications of elevated stress levels – such as increased morning cortisol concentration and nocturnal HR – were observed following the IRS session. On the other hand, increased muscle soreness was observed only in the CON-group who did not engage in post-exercise IRS. In the final week of the intervention, cortisol was less responsive to the exercise session and recovery, indicating a blunting of physiological responses to a standardized stressor due to the jumping exercises alone or its combination with IRS.

In the first week of the intervention, next-morning saliva cortisol increased to a greater extent after the exercise session in the IRS versus the CON group. In addition, nocturnal HR was also higher in the IRS group after the first exercise session. However, no changes over time in HR and HRV were observed, showing no clear effect of the exercise session with or without IRS in the ANS function. These results are reasonably consistent with the relatively limited evidence available covering stress responses to post-exercise IRS. In a previous study on male basketball players, plasma cortisol also increased 14 hours post-exercise, but nocturnal HR and HRV did not change following IRS compared to passive recovery [1]. Furthermore, firefighters had an elevated HR and inferior HRV immediately after a post-exercise IRS session [45], and similar responses were observed within basketball players during post-exercise IRS [1].

We can also compare our observations with responses to traditional sauna bathing, with some caveats. Traditional saunas heat the occupant by convection, whereas IRS transmits heat predominantly through radiation [46]. In contrast to our findings, traditional sauna bathing performed after endurance or resistance exercise did not increase cortisol concentrations [47], but has been observed to increase HR and shift cardiovascular control from vagal to more sympathetic dominance [14,16]. However, these changes have predominantly been observed during the heat exposure. By 15–30 minutes after traditional sauna bathing, vagal tone was returned to baseline levels [14] or increased beyond baseline [48]. Thus, our study further confirm that post-exercise heat exposure does not appear to have a lasting effect on acute ANS responses beyond those observed during the immediate exposure.

It is known that stress hormone responses to an exercise session diminish after regular training [9]. In addition, regular heat exposure might attenuate cortisol reactivity to exercise [11,12]. In the current study, we observed a decrease in the magnitude of cortisol responses from the first week of the intervention to the sixth week in both groups, showing a decreased neuroendocrine response to post-exercise heating. Thus, it might be that the athletes adapted to the IRS exposure and/or the training, diminishing stress hormone reactivity and indicating that post-exercise IRS is unlikely to increase long-term allostatic load for the athlete. Additionally, we did not observe differences in nocturnal HR and HRV between the first and last week of the intervention. Cardiac autonomic adaptations to long-term interventions may be influenced by the total volume of heat stress [16]. In our study, the post-exercise passive heat exposure stimulus was likely fairly mild compared to a previous study that observed enhanced parasympathetic activity following training in heat [17].

To correctly interpret our response, we should be aware that the ANS (i.e. HR and HRV) and HPA (i.e. cortisol) responses are driven by separate but concordant physiological mechanisms. The ANS responses are neural in nature and occur within milliseconds of exposure to an acute stressor, returning to baseline rapidly upon withdrawal of the stressor [49]. The HPA axis, on the other hand, represents a three-step hormonal cascade, resulting in the release of cortisol from the adrenal glands; a process which takes 20–40 minutes to cortisol peak [50]. Thus, ANS responses to brief stressors typically taking place during the stressor whereas cortisol peaks following its resolution and serves to restore homeostasis [49], which might explain why we observed a time effect in cortisol concentrations but not in HRV.

The second reason why we did not observe changes in the ANS function could relate to the magnitude of ANS reactivity to resistance training. While resistance exercise has been shown to impair HRV during the immediate recovery phase (5–75 min), HRV may not reflect stress induced by resistance exercise during the later stages of recovery (24–48 h), despite declines in physical performance and subjective measures [51]. However, studies on the ANS responses to resistance training report conflicting response. Some studies have reported that resistance training can shift autonomic balance toward sympathetic activation for up to 24 hours post-exercise [52,53]. These studies, however, prescribed a greater degree of overload compared to the present study, such as maximal resistance exercise sessions or 6-day overload strength training period. Conversely, a hypertrophic exercise session performed to failure did not influence the ANS responses during the later stages of recovery [51] and similar responses were observed after training to failure and not to failure [54]. Given the ANS responses to jumping training in the present study, we might suggest that HRV may not be a sufficiently sensitive tool to assess recovery after a broader range of resistance exercise sessions, particularly beyond the first hour of recovery.

In the present study, the CON group, but not the IRS group, reported increased muscle soreness after 36 hours in the first week of the intervention. By the final week of the intervention, there were no differences between groups, demonstrating the participants’ adaptation to the exercise session over the course of the training intervention. Delayed onset muscle soreness is known to occur after unfamiliar and eccentric exercises [55]. Similar results were observed in a previous study from our group, where post-exercise IRS attenuated muscle soreness and improved perceived recovery in male basketball players compared to passive recovery [1]. Similarly, we observed that, overall, perceived recovery was higher in the IRS group. Previous meta-analyses have shown that post-exercise heat can alleviate muscle soreness, but the benefits were only observed with localized heat applications, rather than whole-body heat exposures [56]. Furthermore, post-exercise traditional sauna bathing did not improve perceived recovery [4]. However, an infrared radiating lamp was observed to decrease perceived pain [57]. It has been speculated that infrared heat penetrates deeper under the skin than warm air [46], which may attenuate tissue temperature loss, facilitate muscle circulation and metabolism, and reduce peripheral nerve excitability, and as a result of these factors, alleviate muscle soreness [56]. Thus, future studies should focus to investigate the effects of different heat exposures on subjective measures, considering the potential influence of individual preferences. It should be also noted that subjective measures can be an important moderator in the relationship between training load and performance [58].

We did not find any differences in CMJ between the groups, indicating that the exercise session induced similar levels fatigue in both groups and post-exercise IRS did not improve the immediate recovery of neuromuscular performance. Similar results were found in a previous study which reported no differences in CMJ height following post-exercise IRS (50 °C) or passive recovery [2]. In addition, there were no differences in CMJ performance between traditional sauna or placebo treatment following swimming training [4]. However, IRS attenuated the decrease in CMJ during recovery from a complex resistance exercise session [1]. It is known that increased body temperature, for example after warm-up, improves power production [59], and we could therefore expect that immediately after heat exposure, CMJ performance would have improved in the IRS-group compared to the control group. However, the time spent in the IRS was brief, and body temperature might still have been increased in the control group. Thus, post-exercise heat exposure as brief as the 10-minute IRS session incorporated in the present study might not improve power production during the immediate acute recovery phase.

A major strength of this study was the focus on female participants. Most existing research in this area has included only male participants, but the limited data available on women shows passive sauna bathing may augment women’s neuroendocrine responses to a greater extent than in men [21]. In addition, thermoregulation differs between the sexes because women have, for example, a larger surface area-to-mass ratio, lower sweating capacity, and produce less metabolic heat [60]. However, menstrual cycle and hormonal contraceptive phase might also affect neuroendocrine responses [9], HRV [61], and thermoregulation [22,60]. Based on the menstrual diaries, more than half of the participants in the present study likely performed both exercise sessions in the same phase of menstrual or hormonal cycle. Furthermore, the timeframe utilized to study immediate stress responses was short (2 days) which is likely to limit changes in sex hormones within each experimental trial. However, it is possible that phase shifts within the menstrual or hormonal cycle in individual participants between EX1and EX2 could have influenced body temperature (higher temperatures during the luteal phase and when using combined oral contraceptives) and thermoregulation, thereby contributing to differences in physiological responses. On the other hand, random sampling of female athletes regardless of hormonal contraceptive use and menstrual cycle phase arguably best reflects the real-world conditions of team sports, providing more ecologically valid findings [62]. Additionally, we used athletes as participants because the physiological responses to exercise may differ between recreationally active participants and competitive athletes [23], making the results more applicable to high-performance sport.

A limitation of this study is the small number of participants relative to the variables, such as cortisol, which exhibit high intra-individual variability despite controlled conditions [63]. In future studies, baseline levels of such variables should be based on values over several days. It should also be noted that even though most of the participants rarely sauna bathed (once a month or less) before the intervention, they were not completely unaccustomed to sauna use, which might affect heat-induced physiological responses. However, the participants were not accustomed to IRS use specifically before the intervention. The teams participated in the data collection during the different phases of the competition season, which caused variation in the total training load and thus might have affected stress responses. Future studies should investigate post-exercise heat exposures and their effects on neuroendocrine and ANS responses with a more homogenous study population (for example athletes at the same competitive level) and during a more consistent phase of the training season. Additionally, stress responses should also be examined following different types of exercise sessions, as well as after greater heat exposure (longer duration and higher temperature). In this study, the heat exposure was relatively low, which likely made it insufficient to potentiate training-induced physiological adaptations at the muscle cell level. Moreover, the low thermal load limits the generalizability of the findings to regular sauna users, as typical sauna conditions often involve significantly higher temperatures. Whole-body heat exposures can exert additional stress, especially when combined with a total training load. It is therefore important to investigate a broader range of thermal conditions [5]. However, our study found no evidence of pronounced stress responses after regular use of IRS (10 min, 50 °C) for recovery in a practical setting, including a training session typical of team sports followed by regular, short heat exposure.

## Conclusion

Although heat exposure may have benefits for recovery, there are concerns that it may lead to additional stress, and thus be counterproductive to recovery if used on a regular basis. In the present study, we demonstrated an increased next-morning cortisol response and post-exercise nocturnal heart rate following introduction of IRS for post-exercise recovery in female team sports athletes. However, after six weeks of regular post-exercise IRS, we found no remaining evidence of pronounced stress responses, suggesting that autonomic balance and cortisol response adapted effectively to the new regime. This suggests that regular use of post-exercise heat for recovery is unlikely to increase long-term allostatic load for athletes, although it remains unclear whether IRS is effective to alleviate muscle soreness beyond an initial change in the training regime.

## Abbreviations

ANS: autonomic nervous system
CMJ: counter movement jump
CON: control group
EX1: exercise session 1
EX2: exercise session 2
HF: high frequency power
HPA: hypothalamic – pituitary – adrenal
HR: heart rate
HRV: heart rate variability
IRS: infrared sauna
LF: low frequency power
post: after the exercise session
post-EX: immediately after the exercise session
post-REC: immediately after the recovery procedure
pre: before the exercise session
RMSSD: root mean square of successive differences between normal heartbeats
VLF: very low frequency power

## Supplementary Material

Supplementary materials_Ahokas_Clean.docx

## Data Availability

Data will be made available on reasonable request.
