# Supplementary material for: Salivary cortisol response to post-exercise infrared sauna declines over time
Source: Temperature (Austin). 2025 Apr 27;12(3):281–95. doi: 10.1080/23328940.2025.2493460 (PMC12416168; doi:10.1080/23328940.2025.2493460)
Supplement: Supplementary materials_Ahokas_Clean.docx [file KTMP_A_2493460_SM2579.docx]

SUPPLEMENTARY TABLE 1. The use of hormonal contraceptives, manifestation of amenorrhea (absence of menstruation), oligomenorrhea (infrequent menstrual periods, cycle length >35 days), and cycle phase during the experimental trials. Participants reported days of bleeding and possible symptoms related to the menstrual or hormonal cycle. Furthermore, the diary included questions about the regularity of the menstrual cycle, the use of hormonal contraceptives and dates of the previous menstruation. The cycle was divided in the first and the second half of the cycle based on the length of the cycle and the assumption that the duration of the luteal phase is about 14 days.

|  | IRS | CON |
| --- | --- | --- |
| Naturally menstruating (NM) | 9 | 13 |
| Amenorrheic/ oligomenorrheic (NM only) | 3 | 1 |
| Combined hormonal contraceptives (CHC) | 3 | 4 |
| Progestin-only hormonal contraceptives (PHC) | 4 | 1 |
| 1^st^ and 2^nd^ loading in the same phase of the MC | NM = 5, CHC = 1 | NM = 5, CHC = 4 |
| 1^st^ loading in the 1^st^ phase and 2^nd^ loading in the 2^nd^ phase | NM = 0 | NM = 2 |
| 1^st^ loading in the 2^nd^ phase and 2^nd^ loading in the 1^st^ phase | NM = 1 | NM = 5 |
| 1^st^ loading in the ACT and 2^nd^ loading in the INACT | CHC = 2 | CHC = 0 |
| 1^st^ loading in the INACT and 2^nd^ loading in the ACT | CHC = 0 | CHC = 0 |
| Diary was not obtained | 4 | 2 |

ACT = active/pill phase in combined hormonal contraceptives’ users; CON = control group; IRS = infrared sauna group; INACT = inactive/pill-free phase in combined hormonal contraceptives’ users; MC = menstrual cycle; PRE = before intervention; POST = after intervention.

SUPPLEMENTARY TABLE 2. Exercises and repetitions (reps) of the training program’s weekly sessions.

| Session | Exercise | Reps |
| --- | --- | --- |
| 1 | 1.1 Squats | 4 |
|  | 1.2 Box jumps | 6 |
|  | 2.1 Land mine curtsy lunges | 4 + 4 |
|  | 2.2 Drop jumps | 6 + 6 |
|  | 3.1 Pull ups/ Lat pull down | 4 |
|  | 3.2 Medicine ball slams | 6 |
| 2 | 1.1 Hip thrusts | 4 |
|  | 1.2 10 m resisted sprint | 4 |
|  | 2.1 Forward lunges | 4 + 4 |
|  | 2.2 Hop run | 6 + 6 |
|  | 3.1 Bench press | 4 |
|  | 3.2 Medicine ball throw | 6 |
| 3 | 1.1 Split squats | 4 |
|  | 1.2 Drop jumps | 4 |
|  | 2.1 Hip thrusts | 4 |
|  | 2.2 Horizontal jumps | 4 |
|  | 3.1 Military press | 4 |
|  | 3.2 Trunk rotation with bar | 6 |

SUPPLEMENTARY TABLE 3. F- and p-values of timepoint and group effects, and group*timepoint interactions analysed with a linear mixed-effects model. Model 1 included exercise trial 1 (EX1; during the first week of the intervention), and model 2 included exercise trial 2 (EX2; during the sixth week of the intervention).

|  |  | Model 1 (EX1) | | Model 2 (EX2) | |
| --- | --- | --- | --- | --- | --- |
|  |  | F-value | p-value | F-value | p-value |
| CMJ (m) | Group | 0.25 | 0.618 | 0.41 | 0.522 |
|  | Timepoint | 1.87 | 0.159 | **3.75** | **0.027** |
|  | G*T interaction | 0.16 | 0.849 | 0.05 | 0.956 |
| log_2_Cortisol | Group | 0.33 | 0.566 | 0.03 | 0.864 |
| (nmol/l) | Timepoint | **6.38** | **0.014** | 0.19 | 0.668 |
|  | G*T interaction | 0.29 | 0.595 | 0.46 | 0.503 |
| HR (1/min) | Group | **9.16** | **0.004** | **4.32** | **0.043** |
|  | Timepoint | 1.12 | 0.295 | 0.21 | 0.650 |
|  | G*T interaction | 0.21 | 0.651 | 0.03 | 0.856 |
| RMSSD (ms) | Group | 0.00 | 0.971 | 0.00 | 0.983 |
|  | Timepoint | 0.29 | 0.591 | 0.39 | 0.534 |
|  | G*T interaction | 0.01 | 0.945 | 0.00 | 0.977 |
| VLF (%) | Group | **8.93** | **0.004** | 2.41 | 0.127 |
|  | Timepoint | 0.44 | 0.508 | 0.02 | 0.889 |
|  | G*T interaction | 0.05 | 0.825 | 0.23 | 0.638 |
| LF (%) | Group | 0.59 | 0.444 | 0.75 | 0.391 |
|  | Timepoint | 0.12 | 0.735 | 0.00 | 0.983 |
|  | G*T interaction | 0.38 | 0.543 | 0.02 | 0.879 |
| HF (%) | Group | 1.96 | 0.166 | 1.32 | 0.257 |
|  | Timepoint | 0.21 | 0.650 | 0.00 | 0.957 |
|  | G*T interaction | 0.33 | 0.566 | 0.00 | 0.989 |
| Muscle soreness | Group | **4.91** | **0.029** | 0.00 | 0.971 |
|  | Timepoint | **3.82** | **0.025** | 2.16 | 0.122 |
|  | G*T interaction | 0.30 | 0.740 | 0.76 | 0.470 |
| Perceived recovery | Group | 0.00 | 0.957 | **4.46** | **0.039** |
|  | Timepoint | 0.65 | 0.425 | 0.41 | 0.525 |
|  | G*T interaction | 0.07 | 0.798 | 0.20 | 0.658 |

*CMJ* countermovement jump; *HF* high frequency power; *HR* heart rate; *LF* low frequency power; *RMSSD* root mean square of successive differences between normal heartbeats; *VLF* very low frequency.

SUPPLEMENTARY TABLE 4. Comparisons of pre-post changes analysed with linear mixed model (F- and p-values of trial and group effects, and group*trial interactions).

|  |  | F-value | p-value |
| --- | --- | --- | --- |
| ΔCMJ (m) | Group | 3.09 | 0.083 |
| postEX-pre | Trial | 0.28 | 0.599 |
|  | G*T interaction | 0.05 | 0.819 |
| ΔCMJ (m) | Group | 3.06 | 0.085 |
| postREC-pre | Trial | 3.16 | 0.080 |
|  | G*T interaction | 0.37 | 0.547 |
| ΔCMJ (m) | Group | 0.20 | 0.658 |
| postREC-postEX | Trial | 2.56 | 0.114 |
|  | G*T interaction | 0.84 | 0.362 |
| Δlog_2_Cortisol (nmol/l) | Group | 0.99 | 0.325 |
| post-pre | Trial | **5.78** | **0.019** |
|  | G*T interaction | 0.02 | 0.897 |
| ΔHR (1/min) | Group | 0.79 | 0.377 |
| post-pre | Trial | 0.55 | 0.461 |
|  | G*T interaction | 0.12 | 0.734 |
| ΔRMSSD (ms) | Group | 0.02 | 0.897 |
| post-pre | Trial | 0.08 | 0.783 |
|  | G*T interaction | 0.00 | 0.967 |
| ΔVLF (%) | Group | 0.17 | 0.679 |
| post-pre | Trial | 1.08 | 0.304 |
|  | G*T interaction | 0.94 | 0.336 |
| ΔLF (%) | Group | 0.96 | 0.332 |
| post-pre | Trial | 0.20 | 0.654 |
|  | G*T interaction | 0.28 | 0.599 |
| ΔHF (%) | Group | 0.58 | 0.449 |
| post-pre | Trial | 0.45 | 0.507 |
|  | G*T interaction | 0.52 | 0.475 |
| ΔMuscle soreness | Group | 1.12 | 0.295 |
| post12h-pre | Trial | 0.85 | 0.360 |
|  | G*T interaction | 0.37 | 0.548 |
| ΔMuscle soreness | Group | 0.18 | 0.671 |
| post36h-pre | Trial | 1.44 | 0.235 |
|  | G*T interaction | 0.15 | 0.698 |
| ΔMuscle soreness | Group | 3.57 | 0.064 |
| post36h-post12h | Trial | 0.20 | 0.658 |
|  | G*T interaction | 0.00 | 0.952 |
| ΔPerceived recovery | Group | 0.20 | 0.660 |
| post-pre | Trial | 0.28 | 0.596 |
|  | G*T interaction | 1.06 | 0.308 |

*CMJ* countermovement jump; *HF* high frequency power; *HR* heart rate; *LF* low frequency power; *pre* before the exercise protocol, *postEX* after the exercise protocol; *postREC* after the recovery method; *post12h* after 12 hours recovery; *post36h* after 36 hours recovery; *RMSSD* root mean square of successive differences between normal heartbeats; *VLF* very low frequency.


Supplementary figure 1. Participant flow chart. Because of missing data and the dropouts, data from some participants was not utilized. If data was missing in a timepoint (pre/post), the data of the participant was not used in any of the timepoint in statistical analyses of physiological and physical performance variables because of the inter-individual variability. Furthermore, if the data was missing in EX1, the participant was not used in analyses of EX2. *CON* control group, *IRS* infrared sauna group, *HR* heart rate, *HRV* heart rate variability, *MS* muscle soreness.


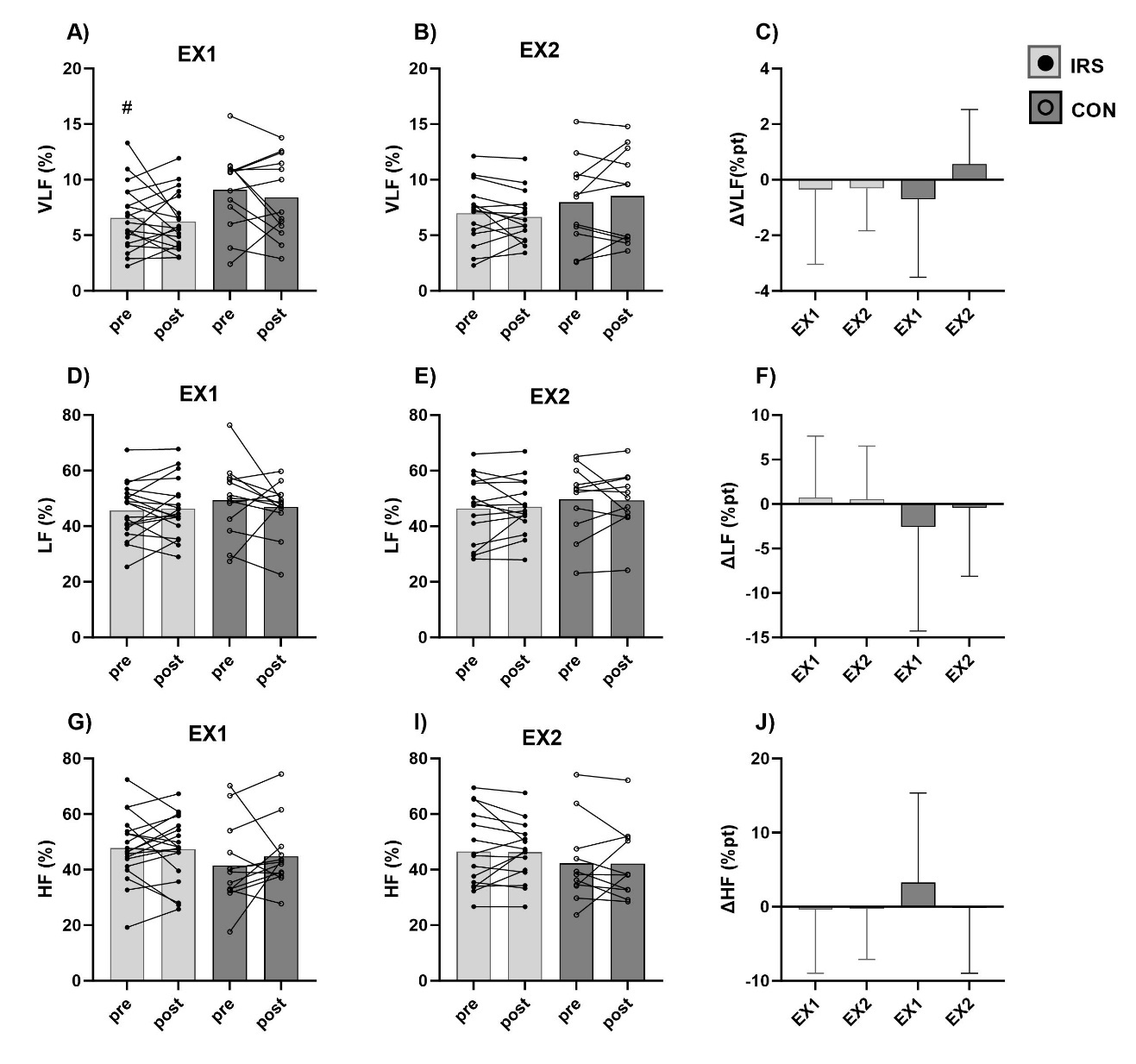

Supplementary figure 2. Nocturnal autonomic nervous system power variables before (pre) and after (post) the exercise loading in the first (EX1; A, D, and G) and in the last week (EX2; B, E, and I) of the training intervention, and changes in response magnitude from EX1 to EX2 within groups (C, F, and J). A-C) very low frequency (VLF) power, D-F) low frequency (LF) power, G-J) high frequency (HF) power. *CON* control group, *IRS* infrared sauna group. # p<0.05 compared to CON-group.
